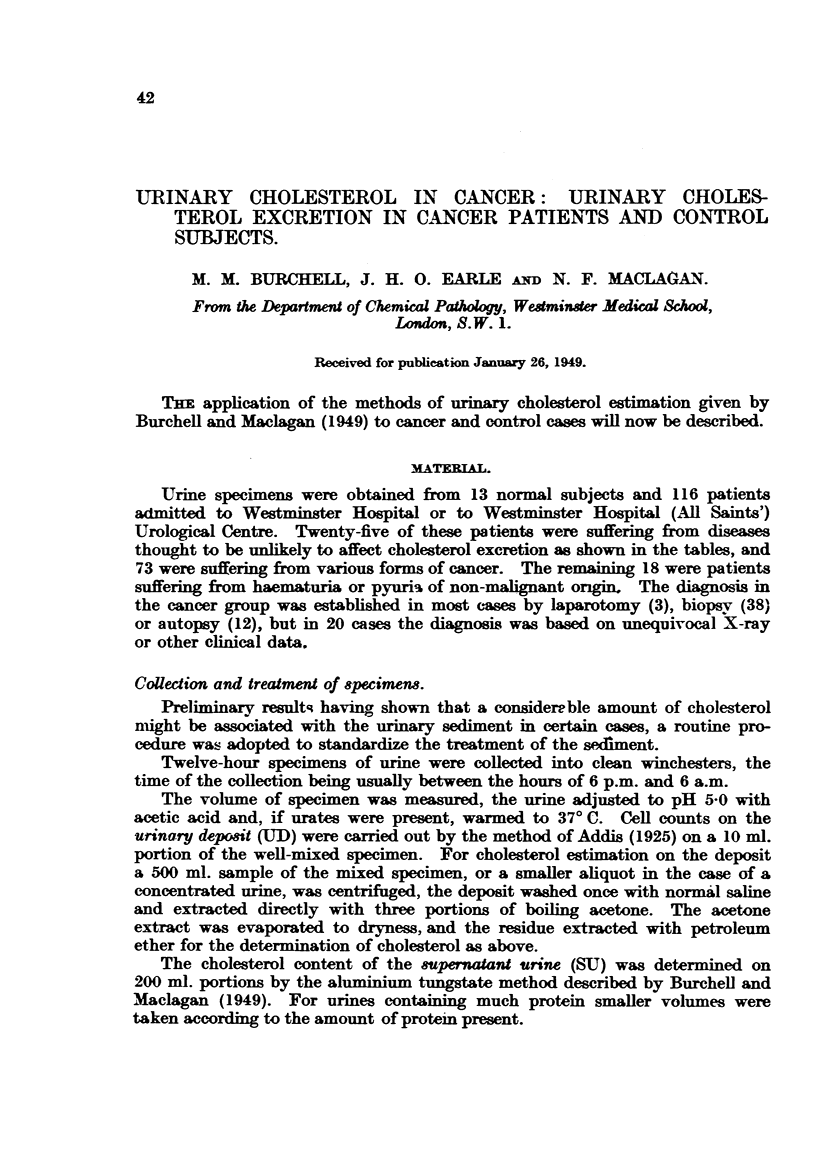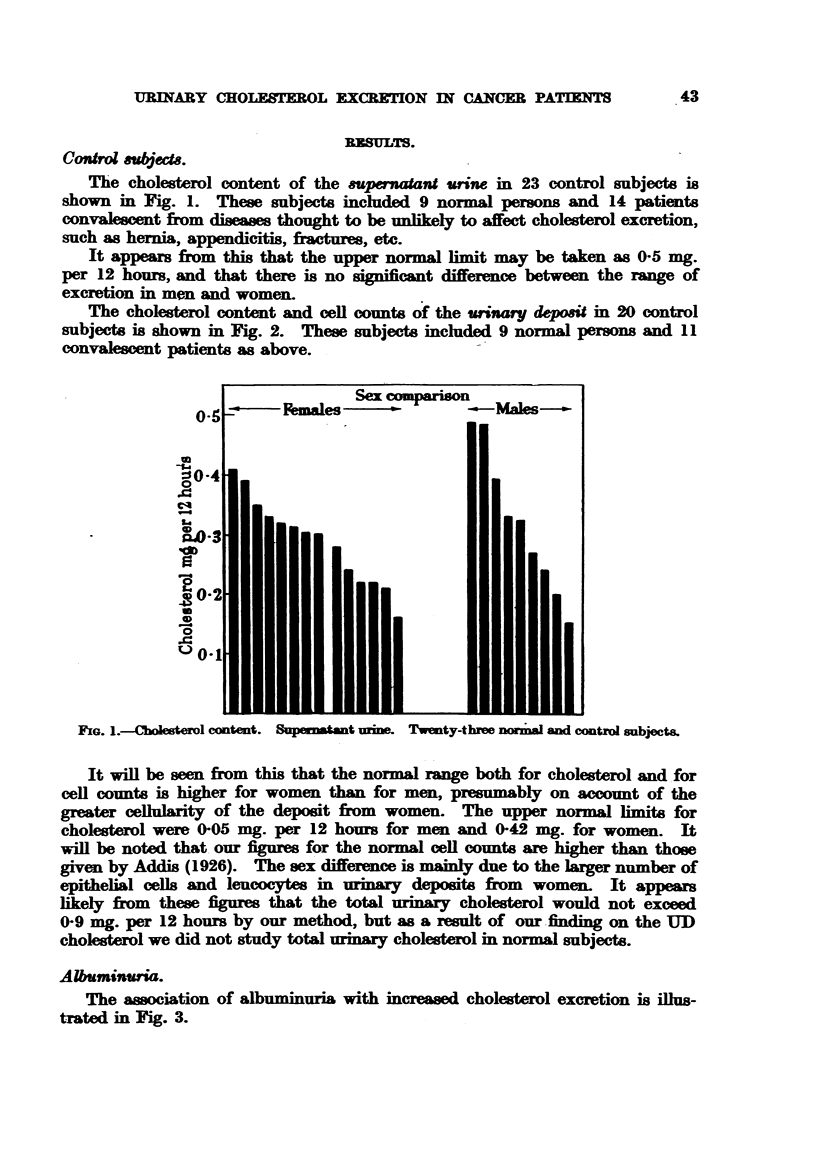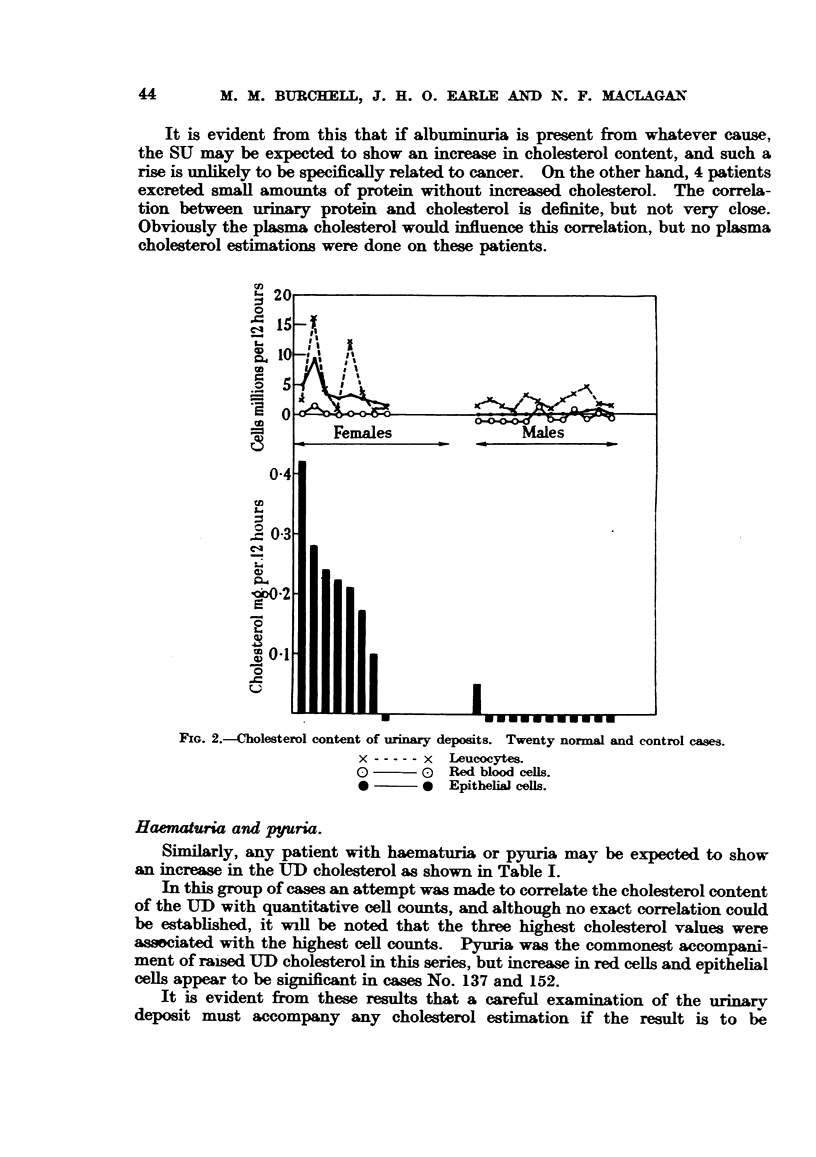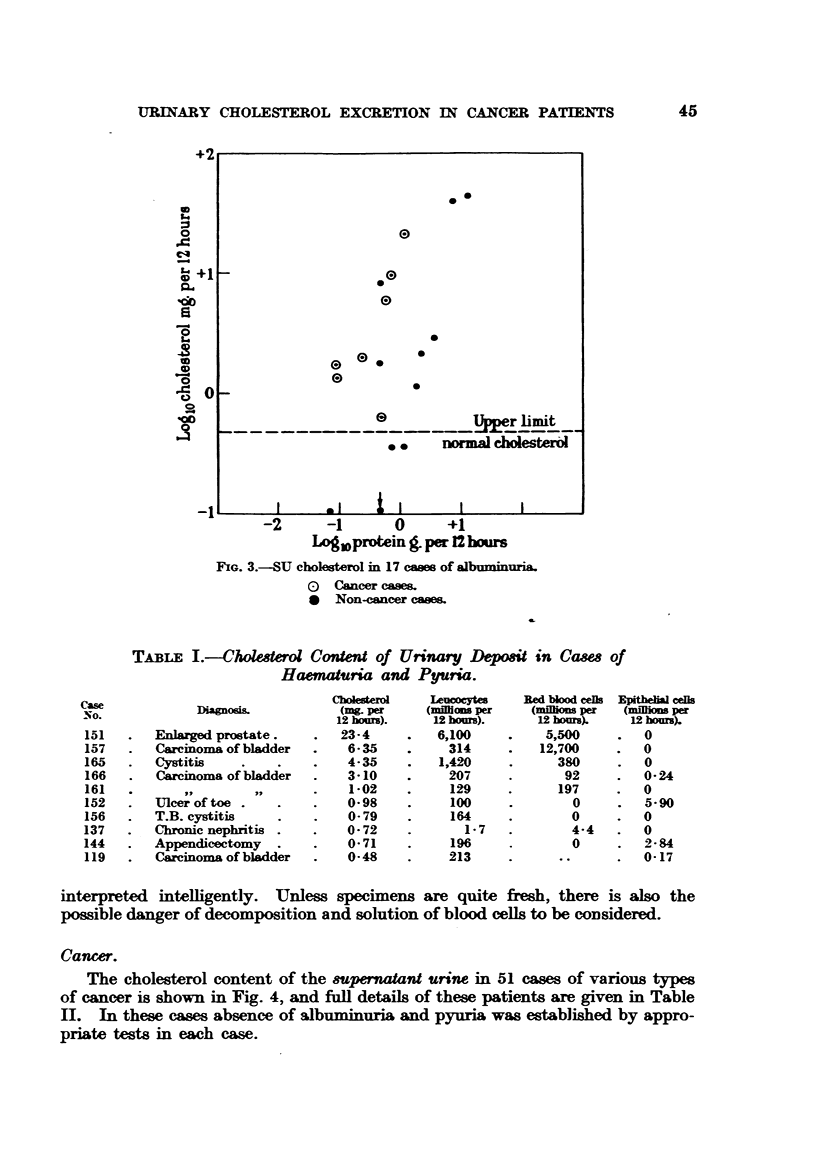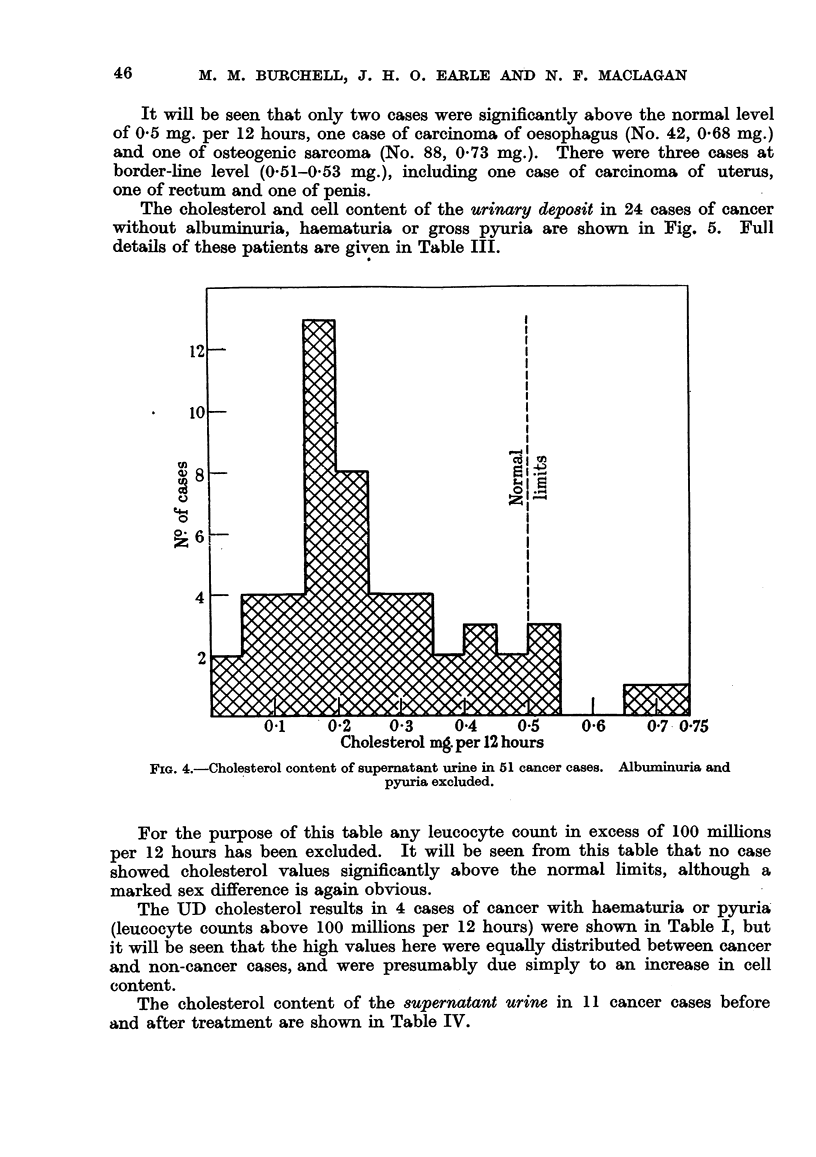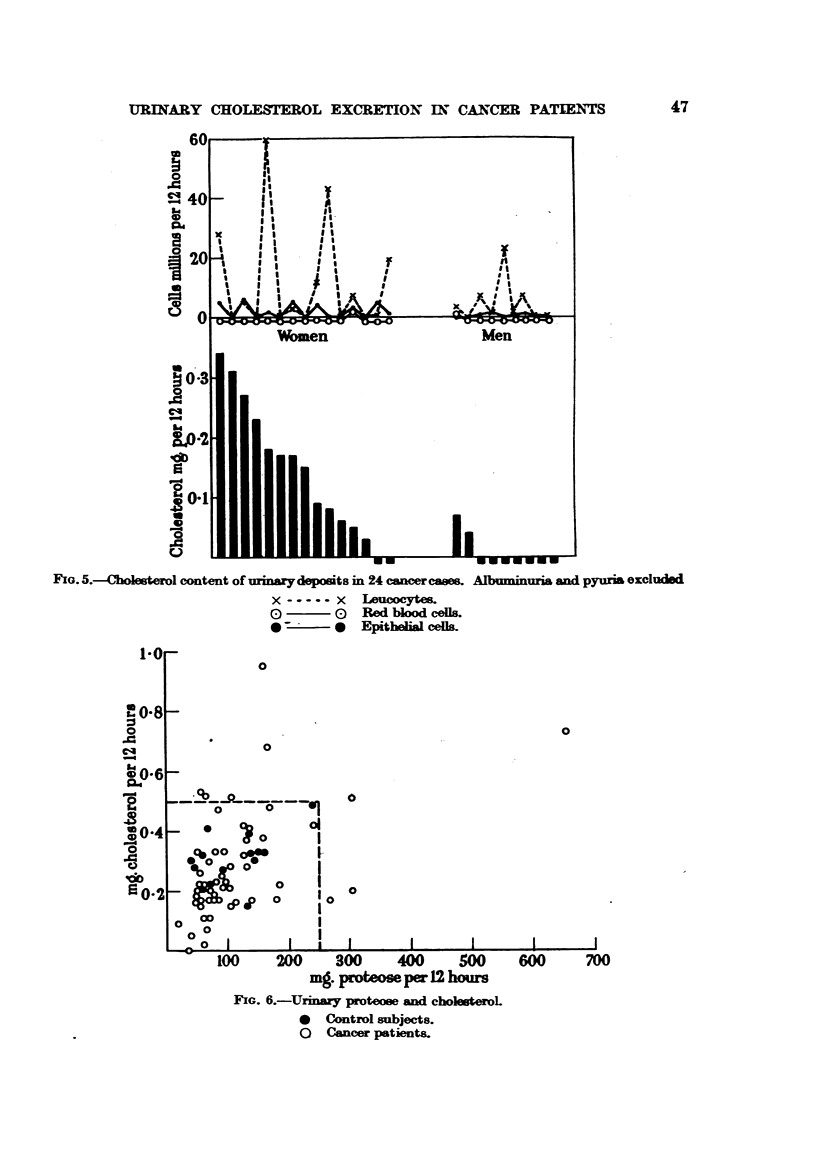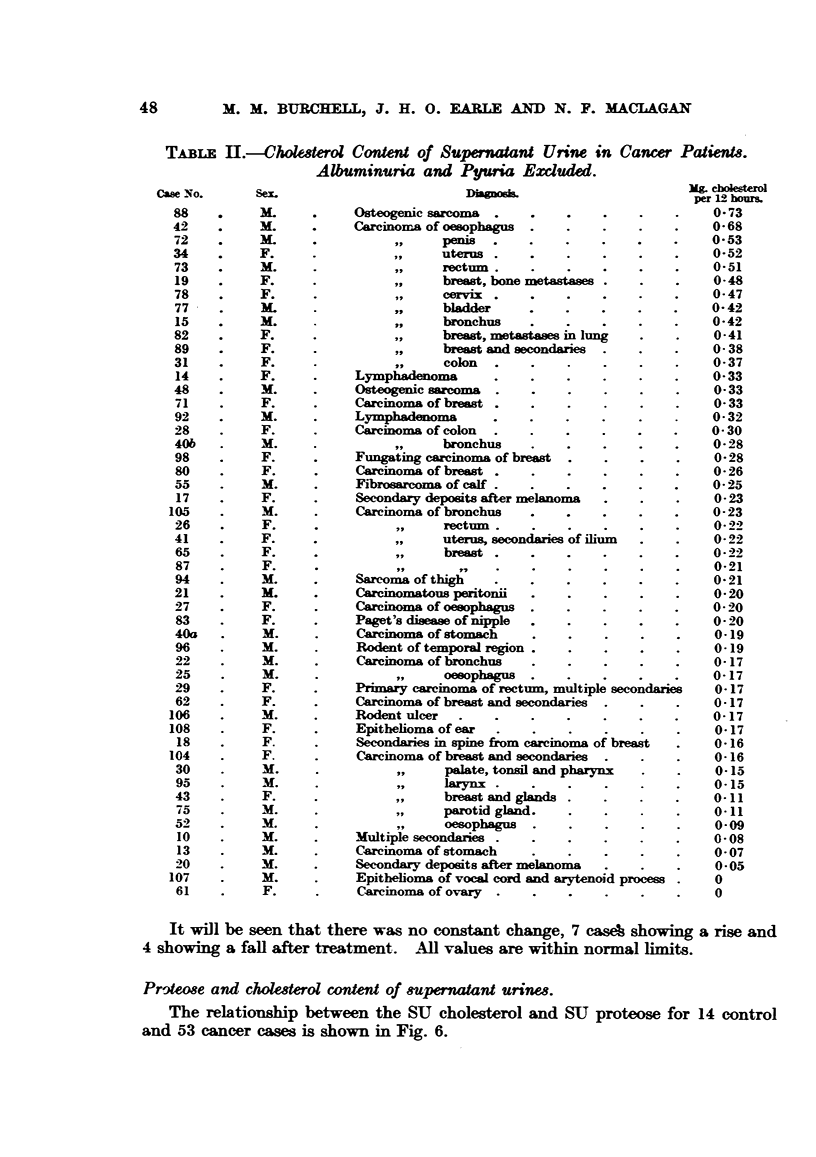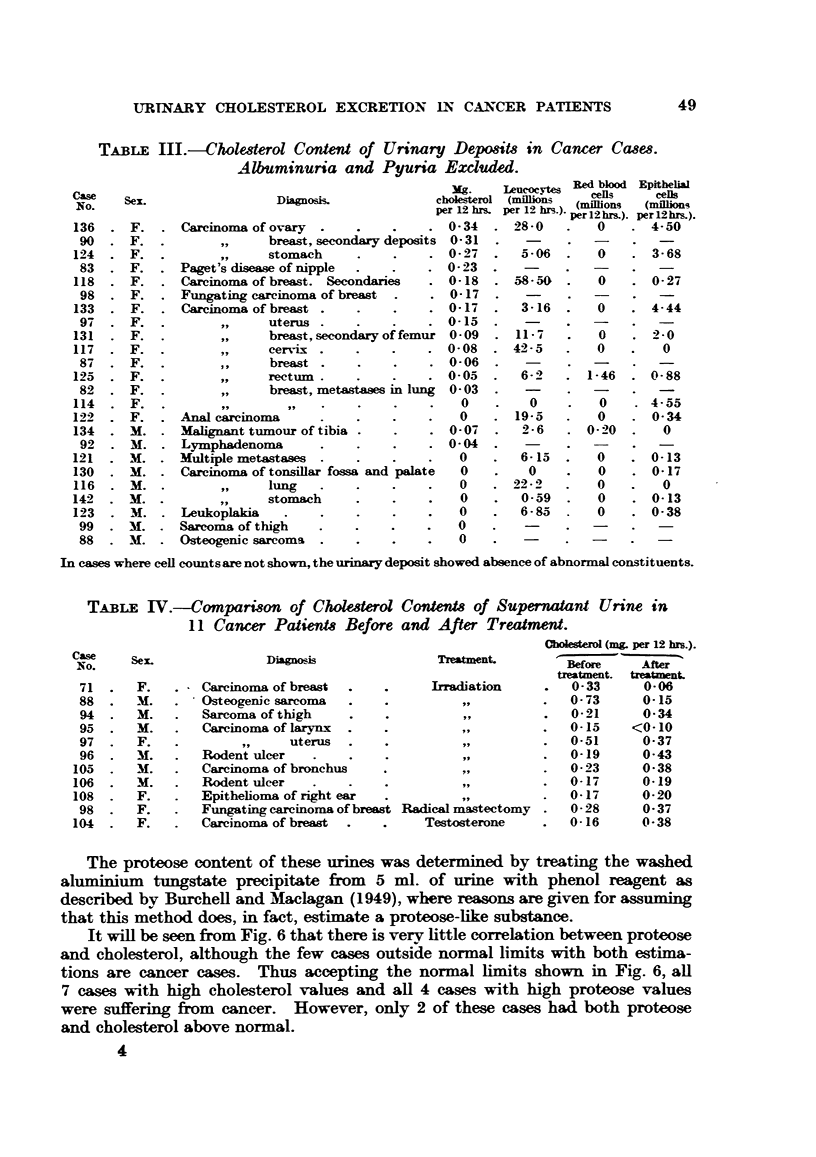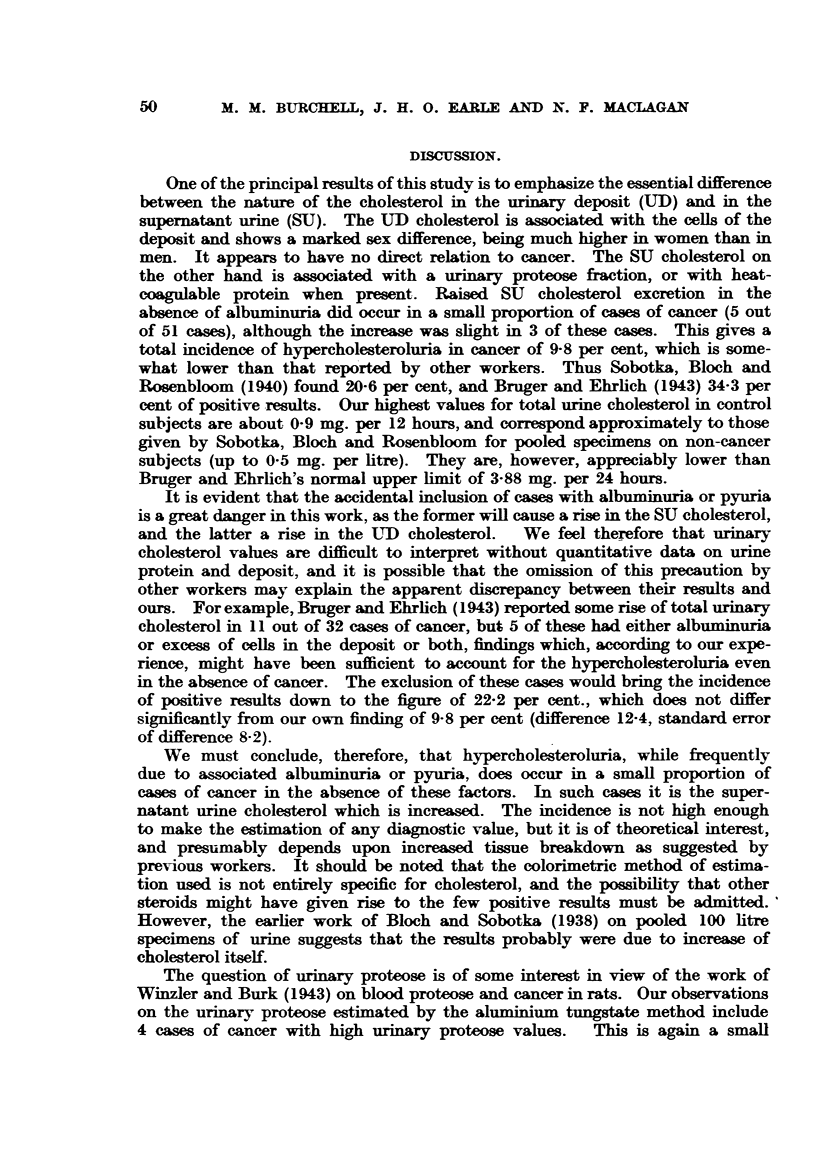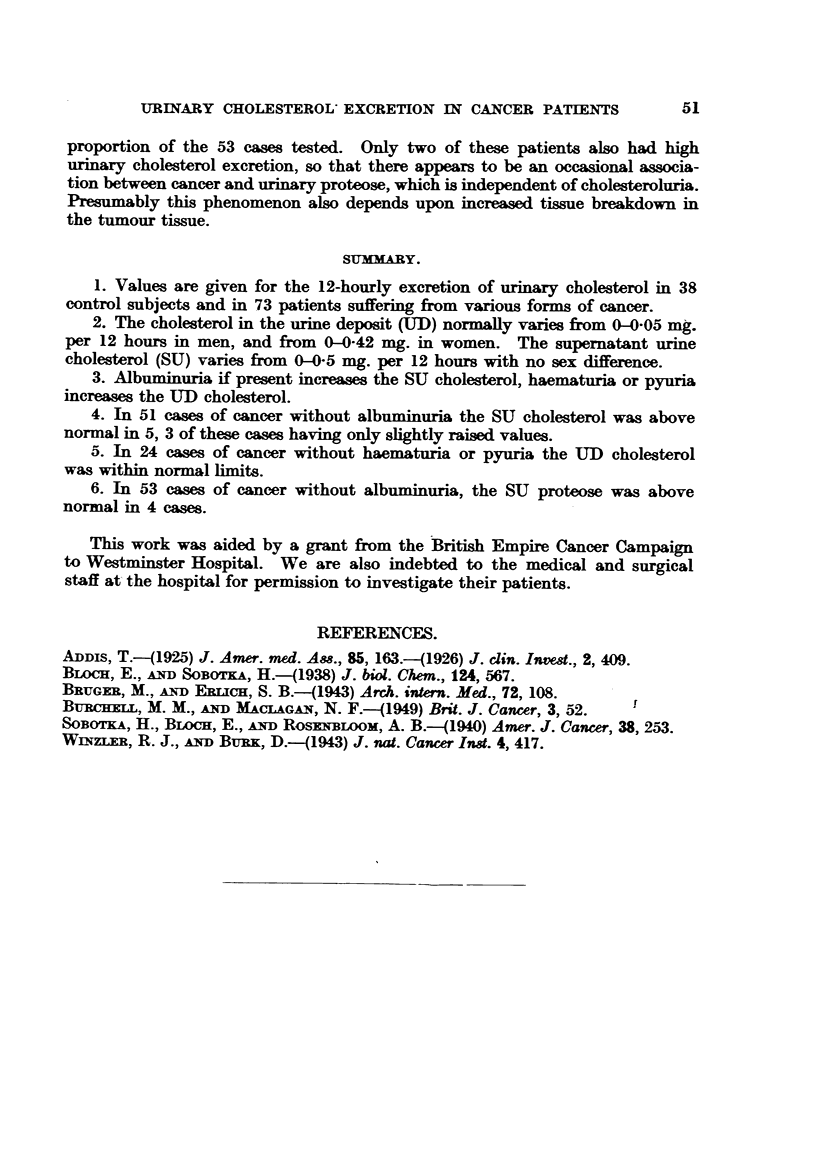# Urinary Cholesterol in Cancer: Urinary Cholesterol Excretion in Cancer Patients and Control Subjects

**Published:** 1949-03

**Authors:** M. M. Burchell, J. H. O. Earle, N. F. Maclagan


### URINARY CHOLESTEROL IN CANCER: URINARY CHOLESTEROL EXCRETION IN CANCER PATIENTS AND CONTROL SUBJECTS.

M. M. BURCHELL, J. H. O. EARLE AND N. F. MACLAGAN.

*From the Department of Chemical Pathology, Westminster Medical School, London, S.W. 1.*

Received for publication January 26, 1949.

THE application of the methods of urinary cholesterol estimation given by Burchell and Maclagan (1949) to cancer and control cases will now be described.

### MATERIAL.

Urine specimens were obtained from 13 normal subjects and 116 patients admitted to Westminster Hospital or to Westminster Hospital (All Saints') Urological Centre. Twenty-five of these patients were suffering from diseases thought to be unlikely to affect cholesterol excretion as shown in the tables, and 73 were suffering from various forms of cancer. The remaining 18 were patients suffering from haematuria or pyuria of non-malignant origin. The diagnosis in the cancer group was established in most cases by laparotomy (3), biopsy (38) or autopsy (12), but in 20 cases the diagnosis was based on unequivocal X-ray or other clinical data.

### *Collection and treatment of specimens.*

Preliminary results having shown that a considerable amount of cholesterol might be associated with the urinary sediment in certain cases, a routine procedure was adopted to standardize the treatment of the sediment.

Twelve-hour specimens of urine were collected into clean winchesters, the time of the collection being usually between the hours of 6 p.m. and 6 a.m.

The volume of specimen was measured, the urine adjusted to pH 5·0 with acetic acid and, if urates were present, warmed to 37° C. Cell counts on the *urinary deposit* (UD) were carried out by the method of Addis (1925) on a 10 ml. portion of the well-mixed specimen. For cholesterol estimation on the deposit a 500 ml. sample of the mixed specimen, or a smaller aliquot in the case of a concentrated urine, was centrifuged, the deposit washed once with normal saline and extracted directly with three portions of boiling acetone. The acetone extract was evaporated to dryness, and the residue extracted with petroleum ether for the determination of cholesterol as above.

The cholesterol content of the *supernatant urine* (SU) was determined on 200 ml. portions by the aluminium tungstate method described by Burchell and Maclagan (1949). For urines containing much protein smaller volumes were taken according to the amount of protein present.

RESULTS.

*Control subjects.*

The cholesterol content of the *supernatant urine* in 23 control subjects is shown in Fig. 1. These subjects included 9 normal persons and 14 patients convalescent from diseases thought to be unlikely to affect cholesterol excretion, such as hernia, appendicitis, fractures, etc.

It appears from this that the upper normal limit may be taken as 0·5 mg. per 12 hours, and that there is no significant difference between the range of excretion in men and women.

The cholesterol content and cell counts of the *urinary deposit* in 20 control subjects is shown in Fig. 2. These subjects included 9 normal persons and 11 convalescent patients as above.

FIG. 1.—Cholesterol content. Supernatant urine. Twenty-three normal and control subjects.

It will be seen from this that the normal range both for cholesterol and for cell counts is higher for women than for men, presumably on account of the greater cellularity of the deposit from women. The upper normal limits for cholesterol were 0·05 mg. per 12 hours for men and 0·42 mg. for women. It will be noted that our figures for the normal cell counts are higher than those given by Addis (1926). The sex difference is mainly due to the larger number of epithelial cells and leucocytes in urinary deposits from women. It appears likely from these figures that the total urinary cholesterol would not exceed 0·9 mg. per 12 hours by our method, but as a result of our finding on the UD cholesterol we did not study total urinary cholesterol in normal subjects.

*Albuminuria.*

The association of albuminuria with increased cholesterol excretion is illustrated in Fig. 3.

It is evident from this that if albuminuria is present from whatever cause, the SU may be expected to show an increase in cholesterol content, and such a rise is unlikely to be specifically related to cancer. On the other hand, 4 patients excreted small amounts of protein without increased cholesterol. The correlation between urinary protein and cholesterol is definite, but not very close. Obviously the plasma cholesterol would influence this correlation, but no plasma cholesterol estimations were done on these patients.

FIG. 2.—Cholesterol content of urinary deposits. Twenty normal and control cases.

× - - - - - × Leucocytes.
⊙ ——— ⊙ Red blood cells.
● ——— ● Epithelial cells.

*Haematuria and pyuria.*

Similarly, any patient with haematuria or pyuria may be expected to show an increase in the UD cholesterol as shown in Table I.

In this group of cases an attempt was made to correlate the cholesterol content of the UD with quantitative cell counts, and although no exact correlation could be established, it will be noted that the three highest cholesterol values were associated with the highest cell counts. Pyuria was the commonest accompaniment of raised UD cholesterol in this series, but increase in red cells and epithelial cells appear to be significant in cases No. 137 and 152.

It is evident from these results that a careful examination of the urinary deposit must accompany any cholesterol estimation if the result is to be

FIG. 3.—SU cholesterol in 17 cases of albuminuria.
⊙ Cancer cases.
● Non-cancer cases.

TABLE I.—*Cholesterol Content of Urinary Deposit in Cases of Haematuria and Pyuria.*

| Case No. | Diagnosis. | Cholesterol (mg. per 12 hours). | Leucocytes (millions per 12 hours). | Red blood cells (millions per 12 hours). | Epithelial cells (millions per 12 hours). |
|---|---|---|---|---|---|
| 151 | Enlarged prostate | 23·4 | 6,100 | 5,500 | 0 |
| 157 | Carcinoma of bladder | 6·35 | 314 | 12,700 | 0 |
| 165 | Cystitis | 4·35 | 1,420 | 380 | 0 |
| 166 | Carcinoma of bladder | 3·10 | 207 | 92 | 0·24 |
| 161 | ,, ,, | 1·02 | 129 | 197 | 0 |
| 152 | Ulcer of toe | 0·98 | 100 | 0 | 5·90 |
| 156 | T.B. cystitis | 0·79 | 164 | 0 | 0 |
| 137 | Chronic nephritis | 0·72 | 1·7 | 4·4 | 0 |
| 144 | Appendicectomy | 0·71 | 196 | 0 | 2·84 |
| 119 | Carcinoma of bladder | 0·48 | 213 | .. | 0·17 |

interpreted intelligently. Unless specimens are quite fresh, there is also the possible danger of decomposition and solution of blood cells to be considered.

*Cancer.*

The cholesterol content of the *supernatant urine* in 51 cases of various types of cancer is shown in Fig. 4, and full details of these patients are given in Table II. In these cases absence of albuminuria and pyuria was established by appropriate tests in each case.

It will be seen that only two cases were significantly above the normal level of 0·5 mg. per 12 hours, one case of carcinoma of oesophagus (No. 42, 0·68 mg.) and one of osteogenic sarcoma (No. 88, 0·73 mg.). There were three cases at border-line level (0·51–0·53 mg.), including one case of carcinoma of uterus, one of rectum and one of penis.

The cholesterol and cell content of the *urinary deposit* in 24 cases of cancer without albuminuria, haematuria or gross pyuria are shown in Fig. 5. Full details of these patients are given in Table III.

Fig. 4.—Cholesterol content of supernatant urine in 51 cancer cases. Albuminuria and pyuria excluded.

For the purpose of this table any leucocyte count in excess of 100 millions per 12 hours has been excluded. It will be seen from this table that no case showed cholesterol values significantly above the normal limits, although a marked sex difference is again obvious.

The UD cholesterol results in 4 cases of cancer with haematuria or pyuria (leucocyte counts above 100 millions per 12 hours) were shown in Table I, but it will be seen that the high values here were equally distributed between cancer and non-cancer cases, and were presumably due simply to an increase in cell content.

The cholesterol content of the *supernatant urine* in 11 cancer cases before and after treatment are shown in Table IV.

FIG. 5.—Cholesterol content of urinary deposits in 24 cancer cases. Albuminuria and pyuria excluded

× - - - - - × Leucocytes.
⊙ ——— ⊙ Red blood cells.
● ——— ● Epithelial cells.

FIG. 6.—Urinary proteose and cholesterol.

● Control subjects.

○ Cancer patients.

TABLE II.—*Cholesterol Content of Supernatant Urine in Cancer Patients. Albuminuria and Pyuria Excluded.*

| Case No. | Sex. | Diagnosis. | Mg. cholesterol per 12 hours. |
|---|---|---|---|
| 88 | M. | Osteogenic sarcoma | 0·73 |
| 42 | M. | Carcinoma of oesophagus | 0·68 |
| 72 | M. | ,, penis | 0·53 |
| 34 | F. | ,, uterus | 0·52 |
| 73 | M. | ,, rectum | 0·51 |
| 19 | F. | ,, breast, bone metastases | 0·48 |
| 78 | F. | ,, cervix | 0·47 |
| 77 | M. | ,, bladder | 0·42 |
| 15 | M. | ,, bronchus | 0·42 |
| 82 | F. | ,, breast, metastases in lung | 0·41 |
| 89 | F. | ,, breast and secondaries | 0·38 |
| 31 | F. | ,, colon | 0·37 |
| 14 | F. | Lymphadenoma | 0·33 |
| 48 | M. | Osteogenic sarcoma | 0·33 |
| 71 | F. | Carcinoma of breast | 0·33 |
| 92 | M. | Lymphadenoma | 0·32 |
| 28 | F. | Carcinoma of colon | 0·30 |
| 40*b* | M. | ,, bronchus | 0·28 |
| 98 | F. | Fungating carcinoma of breast | 0·28 |
| 80 | F. | Carcinoma of breast | 0·26 |
| 55 | M. | Fibrosarcoma of calf | 0·25 |
| 17 | F. | Secondary deposits after melanoma | 0·23 |
| 105 | M. | Carcinoma of bronchus | 0·23 |
| 26 | F. | ,, rectum | 0·22 |
| 41 | F. | ,, uterus, secondaries of ilium | 0·22 |
| 65 | F. | ,, breast | 0·22 |
| 87 | F. | ,, ,, | 0·21 |
| 94 | M. | Sarcoma of thigh | 0·21 |
| 21 | M. | Carcinomatous peritonii | 0·20 |
| 27 | F. | Carcinoma of oesophagus | 0·20 |
| 83 | F. | Paget's disease of nipple | 0·20 |
| 40*a* | M. | Carcinoma of stomach | 0·19 |
| 96 | M. | Rodent of temporal region | 0·19 |
| 22 | M. | Carcinoma of bronchus | 0·17 |
| 25 | M. | ,, oesophagus | 0·17 |
| 29 | F. | Primary carcinoma of rectum, multiple secondaries | 0·17 |
| 62 | F. | Carcinoma of breast and secondaries | 0·17 |
| 106 | M. | Rodent ulcer | 0·17 |
| 108 | F. | Epithelioma of ear | 0·17 |
| 18 | F. | Secondaries in spine from carcinoma of breast | 0·16 |
| 104 | F. | Carcinoma of breast and secondaries | 0·16 |
| 30 | M. | ,, palate, tonsil and pharynx | 0·15 |
| 95 | M. | ,, larynx | 0·15 |
| 43 | F. | ,, breast and glands | 0·11 |
| 75 | M. | ,, parotid gland. | 0·11 |
| 52 | M. | ,, oesophagus | 0·09 |
| 10 | M. | Multiple secondaries | 0·08 |
| 13 | M. | Carcinoma of stomach | 0·07 |
| 20 | M. | Secondary deposits after melanoma | 0·05 |
| 107 | M. | Epithelioma of vocal cord and arytenoid process | 0 |
| 61 | F. | Carcinoma of ovary | 0 |

It will be seen that there was no constant change, 7 cases showing a rise and 4 showing a fall after treatment. All values are within normal limits.

*Proteose and cholesterol content of supernatant urines.*

The relationship between the SU cholesterol and SU proteose for 14 control and 53 cancer cases is shown in Fig. 6.

TABLE III.—*Cholesterol Content of Urinary Deposits in Cancer Cases. Albuminuria and Pyuria Excluded.*

| Case No. | Sex. | Diagnosis. | Mg. cholesterol per 12 hrs. | Leucocytes (millions per 12 hrs.). | Red blood cells (millions per 12 hrs.). | Epithelial cells (millions per 12 hrs.). |
|---|---|---|---|---|---|---|
| 136 | F. | Carcinoma of ovary | 0·34 | 28·0 | 0 | 4·50 |
| 90 | F. | ,, breast, secondary deposits | 0·31 | — | — | — |
| 124 | F. | ,, stomach | 0·27 | 5·06 | 0 | 3·68 |
| 83 | F. | Paget's disease of nipple | 0·23 | — | — | — |
| 118 | F. | Carcinoma of breast. Secondaries | 0·18 | 58·50 | 0 | 0·27 |
| 98 | F. | Fungating carcinoma of breast | 0·17 | — | — | — |
| 133 | F. | Carcinoma of breast | 0·17 | 3·16 | 0 | 4·44 |
| 97 | F. | ,, uterus | 0·15 | — | — | — |
| 131 | F. | ,, breast, secondary of femur | 0·09 | 11·7 | 0 | 2·0 |
| 117 | F. | ,, cervix | 0·08 | 42·5 | 0 | 0 |
| 87 | F. | ,, breast | 0·06 | — | — | — |
| 125 | F. | ,, rectum | 0·05 | 6·2 | 1·46 | 0·88 |
| 82 | F. | ,, breast, metastases in lung | 0·03 | — | — | — |
| 114 | F. | ,, ,, | 0 | 0 | 0 | 4·55 |
| 122 | F. | Anal carcinoma | 0 | 19·5 | 0 | 0·34 |
| 134 | M. | Malignant tumour of tibia | 0·07 | 2·6 | 0·20 | 0 |
| 92 | M. | Lymphadenoma | 0·04 | — | — | — |
| 121 | M. | Multiple metastases | 0 | 6·15 | 0 | 0·13 |
| 130 | M. | Carcinoma of tonsillar fossa and palate | 0 | 0 | 0 | 0·17 |
| 116 | M. | ,, lung | 0 | 22·2 | 0 | 0 |
| 142 | M. | ,, stomach | 0 | 0·59 | 0 | 0·13 |
| 123 | M. | Leukoplakia | 0 | 6·85 | 0 | 0·38 |
| 99 | M. | Sarcoma of thigh | 0 | — | — | — |
| 88 | M. | Osteogenic sarcoma | 0 | — | — | — |

In cases where cell counts are not shown, the urinary deposit showed absence of abnormal constituents.

TABLE IV.—*Comparison of Cholesterol Contents of Supernatant Urine in 11 Cancer Patients Before and After Treatment.*

| Case No. | Sex. | Diagnosis | Treatment. | Cholesterol (mg. per 12 hrs.). Before treatment. | After treatment. |
|---|---|---|---|---|---|
| 71 | F. | Carcinoma of breast | Irradiation | 0·33 | 0·06 |
| 88 | M. | Osteogenic sarcoma | ,, | 0·73 | 0·15 |
| 94 | M. | Sarcoma of thigh | ,, | 0·21 | 0·34 |
| 95 | M. | Carcinoma of larynx | ,, | 0·15 | <0·10 |
| 97 | F. | ,, uterus | ,, | 0·51 | 0·37 |
| 96 | M. | Rodent ulcer | ,, | 0·19 | 0·43 |
| 105 | M. | Carcinoma of bronchus | ,, | 0·23 | 0·38 |
| 106 | M. | Rodent ulcer | ,, | 0·17 | 0·19 |
| 108 | F. | Epithelioma of right ear | ,, | 0·17 | 0·20 |
| 98 | F. | Fungating carcinoma of breast | Radical mastectomy | 0·28 | 0·37 |
| 104 | F. | Carcinoma of breast | Testosterone | 0·16 | 0·38 |

The proteose content of these urines was determined by treating the washed aluminium tungstate precipitate from 5 ml. of urine with phenol reagent as described by Burchell and Maclagan (1949), where reasons are given for assuming that this method does, in fact, estimate a proteose-like substance.

It will be seen from Fig. 6 that there is very little correlation between proteose and cholesterol, although the few cases outside normal limits with both estimations are cancer cases. Thus accepting the normal limits shown in Fig. 6, all 7 cases with high cholesterol values and all 4 cases with high proteose values were suffering from cancer. However, only 2 of these cases had both proteose and cholesterol above normal.

### DISCUSSION.

One of the principal results of this study is to emphasize the essential difference between the nature of the cholesterol in the urinary deposit (UD) and in the supernatant urine (SU). The UD cholesterol is associated with the cells of the deposit and shows a marked sex difference, being much higher in women than in men. It appears to have no direct relation to cancer. The SU cholesterol on the other hand is associated with a urinary proteose fraction, or with heat-coagulable protein when present. Raised SU cholesterol excretion in the absence of albuminuria did occur in a small proportion of cases of cancer (5 out of 51 cases), although the increase was slight in 3 of these cases. This gives a total incidence of hypercholesteroluria in cancer of 9·8 per cent, which is somewhat lower than that reported by other workers. Thus Sobotka, Bloch and Rosenbloom (1940) found 20·6 per cent, and Bruger and Ehrlich (1943) 34·3 per cent of positive results. Our highest values for total urine cholesterol in control subjects are about 0·9 mg. per 12 hours, and correspond approximately to those given by Sobotka, Bloch and Rosenbloom for pooled specimens on non-cancer subjects (up to 0·5 mg. per litre). They are, however, appreciably lower than Bruger and Ehrlich's normal upper limit of 3·88 mg. per 24 hours.

It is evident that the accidental inclusion of cases with albuminuria or pyuria is a great danger in this work, as the former will cause a rise in the SU cholesterol, and the latter a rise in the UD cholesterol. We feel therefore that urinary cholesterol values are difficult to interpret without quantitative data on urine protein and deposit, and it is possible that the omission of this precaution by other workers may explain the apparent discrepancy between their results and ours. For example, Bruger and Ehrlich (1943) reported some rise of total urinary cholesterol in 11 out of 32 cases of cancer, but 5 of these had either albuminuria or excess of cells in the deposit or both, findings which, according to our experience, might have been sufficient to account for the hypercholesteroluria even in the absence of cancer. The exclusion of these cases would bring the incidence of positive results down to the figure of 22·2 per cent., which does not differ significantly from our own finding of 9·8 per cent (difference 12·4, standard error of difference 8·2).

We must conclude, therefore, that hypercholesteroluria, while frequently due to associated albuminuria or pyuria, does occur in a small proportion of cases of cancer in the absence of these factors. In such cases it is the supernatant urine cholesterol which is increased. The incidence is not high enough to make the estimation of any diagnostic value, but it is of theoretical interest, and presumably depends upon increased tissue breakdown as suggested by previous workers. It should be noted that the colorimetric method of estimation used is not entirely specific for cholesterol, and the possibility that other steroids might have given rise to the few positive results must be admitted. However, the earlier work of Bloch and Sobotka (1938) on pooled 100 litre specimens of urine suggests that the results probably were due to increase of cholesterol itself.

The question of urinary proteose is of some interest in view of the work of Winzler and Burk (1943) on blood proteose and cancer in rats. Our observations on the urinary proteose estimated by the aluminium tungstate method include 4 cases of cancer with high urinary proteose values. This is again a small proportion of the 53 cases tested. Only two of these patients also had high urinary cholesterol excretion, so that there appears to be an occasional association between cancer and urinary proteose, which is independent of cholesteroluria. Presumably this phenomenon also depends upon increased tissue breakdown in the tumour tissue.

### SUMMARY.

1. Values are given for the 12-hourly excretion of urinary cholesterol in 38 control subjects and in 73 patients suffering from various forms of cancer.

2. The cholesterol in the urine deposit (UD) normally varies from 0–0·05 mg. per 12 hours in men, and from 0–0·42 mg. in women. The supernatant urine cholesterol (SU) varies from 0–0·5 mg. per 12 hours with no sex difference.

3. Albuminuria if present increases the SU cholesterol, haematuria or pyuria increases the UD cholesterol.

4. In 51 cases of cancer without albuminuria the SU cholesterol was above normal in 5, 3 of these cases having only slightly raised values.

5. In 24 cases of cancer without haematuria or pyuria the UD cholesterol was within normal limits.

6. In 53 cases of cancer without albuminuria, the SU proteose was above normal in 4 cases.

This work was aided by a grant from the British Empire Cancer Campaign to Westminster Hospital. We are also indebted to the medical and surgical staff at the hospital for permission to investigate their patients.

### REFERENCES.

ADDIS, T.—(1925) *J. Amer. med. Ass.*, **85**, 163.—(1926) *J. clin. Invest.*, **2**, 409.

BLOCH, E., AND SOBOTKA, H.—(1938) *J. biol. Chem.*, **124**, 567.

BRUGER, M., AND ERLICH, S. B.—(1943) *Arch. intern. Med.*, **72**, 108.

BURCHELL, M. M., AND MACLAGAN, N. F.—(1949) *Brit. J. Cancer*, **3**, 52.

SOBOTKA, H., BLOCH, E., AND ROSENBLOOM, A. B.—(1940) *Amer. J. Cancer*, **38**, 253.

WINZLER, R. J., AND BURK, D.—(1943) *J. nat. Cancer Inst.* **4**, 417.